# ^1^H NMR Spectroscopy to Characterize Italian Extra Virgin Olive Oil Blends, Using Statistical Models and Databases Based on Monocultivar Reference Oils

**DOI:** 10.3390/foods9121797

**Published:** 2020-12-03

**Authors:** Chiara Roberta Girelli, Francesca Calò, Federica Angilè, Lucia Mazzi, Daniele Barbini, Francesco Paolo Fanizzi

**Affiliations:** 1 Department of Biological and Environmental Sciences and Technologies, University of Salento, Prov.le Lecce-Monteroni, 73100 Lecce, Italy; chiara.girelli@unisalento.it (C.R.G.); francesca.calo@unisalento.it (F.C.); federica.angile@unisalento.it (F.A.); 2 Certified Origins Italia Srl, Località il Madonnino, 58100 Grosseto, Italy; lucia.mazzi@oleificioolma.it (L.M.); daniele.barbini@certifiedorigins.it (D.B.)

**Keywords:** ^1^H-NMR spectroscopy, extra virgin olive oil, chemometrics, quality, traceability

## Abstract

During the last few years, the global demand for extra virgin olive oil (EVOO) is increased. Olive oil represents a significant percentage of world fat consumption determining an important development of its market. In this context, the problems related to counterfeiting and product fraud is becoming extremely relevant. Thus, the quality and authenticity control of EVOOs is nowadays mandatory. In this study we focused on the use of ^1^H NMR technique associated with multivariate statistical analysis to characterize Italian EVOOs commercial blends. In particular, a specific database including 126 monocultivar EVOOs reference samples, was used to characterize a total of 241 Italian EVOOs blends over four consecutive harvesting years. Moreover, the effect of the minor components (phenolic compounds) on the qualitative characterization of blended EVOOs was also evaluated. The correlation analysis of classification scores obtained using two pairwise orthogonal partial least square-discriminant analysis models (built with major and combined major–minor components NMR data) revealed that both could be profitably used to generally classify the studied Coratina containing blends.

## 1. Introduction

To date, due to its sensorial, nutraceutical, and other well-known health properties, extra virgin olive oil (EVOO) is considered one of the most important classic products of the Mediterranean countries, often called “the yellow gold” [1,2,3]. World olive oil production is still essentially concentrated in the Mediterranean basin, in particular in Spain and Italy, two countries representing almost all world exports (60% Spain and 20% Italy). Italian product covers on average 15% of world production (compared to 45% of Spain) [4]. Italian EVOO is worldwide recognized as a high value product thanks to its well established “health appeal”, quantitatively limited production and organoleptic characteristics [1,5]. Indeed, Italian food market is widespread known for its richness of varieties and high quality products [6]. Over the years, the high national and international commercial value of Italian EVOOs has led to their adulteration with low quality foreign olive oils and in some cases, also with the addition of other low cost edible vegetable oils of uncertain origin. Therefore, the establishment and systematic implementation of a reliable quality control methodology for certifying the EVOO authenticity is a priority issue. The European Union Regulation 182 of 6 March 2009 [7], declared the compulsory labelling of extra virgin olive oils in all European countries with a clear indication of the geographical origin of the olives used for the production. More recently, the Commission Implementing Regulation (EU) No 29/2012 of 13 January 2012 on marketing standards for olive oil stated “As a result of agricultural traditions and local extraction and blending practices directly marketable virgin olive oils may be of quite different taste and quality depending on their geographical origin” and “The experience gained by operators and administrations in this matter allowed making the labelling of the origin compulsory for extra virgin and virgin olive oil” [8]. These regulations aim to ensure transparency for consumers and their purchasing choices, but still lack of an official validated methodology for EVOOs geographical origin assessment. In this respect, an approach aimed at obtaining a fingerprinting of extra virgin olive oil could be useful to ensure product transparency and traceability. Indeed EVOO chemical components profile depends not only on the used olive cultivars but also on the specific geographical areas of origin [9]. As already reported in the literature [10,11,12,13,14,15,16,17,18,19,20,21,22] the ^1^H Nuclear Magnetic Resonance (NMR)-based metabolomic approach represents a powerful tool for EVOOs origin and authenticity assessment. (NMR) is often used to analyze foodstuff and, in particular, olive oil, since it allows to detect major and minor components, giving full account for the natural chemical composition variability of complex matrices. NMR spectroscopy data, associated with multivariate statistics analysis (MVA), increases the prediction efficiency of specific clustering that are usually observed according to EVOOs cultivars and/or geographical origin. Moreover, this technique allows to define EVOOs metabolic profiles accounting for different parameters, such as pedoclimatic conditions (temperature and humidity), growing areas, and agricultural practices [13,23].

Commercial EVOOs generally are blended with oils from many different cultivars and regions, sometimes even from different countries, in order to meet the demands of the market. Consequently the possibility to discriminate different EVOOs is a hot topic also to prevent international frauds [24,25,26]. In Italy reasonably priced 100% Italian EVOO is usually commercialized as Coratina oil blended with other “sweeteners” cultivars [11]. Indeed, due to its richness in phenolic compounds, Coratina oil is markedly bitter and pungent. Therefore, despite his high nutraceutic value, it usually needs to be smoothed with other cultivars EVOOs to become an average consumer accepted commercial blend [12].

In this work we focused on the building of a specific ^1^H NMR profiling database using Coratina oil samples (from Apulia region, South East Italy) and the samples of typical sweeteners cultivars Cima di Mola, Ogliarola barese, Cellina di Nardò (Apulia) together with Rossanese and Carolea (from Calabria region, South Italy). Ogliarola Barese and Cima di Mola are, with Coratina, the most popular olive cultivars in the Bari Province (Apulia, Region) as they are the basis of “Terra di Bari” Protected Designation of Origin (PDO) production [27]. On the other hand, Cellina di Nardò is one of the representative olive cultivars in the Jonian-Salento area (Apulia Region) and, together with Ogliarola salentina is known as “Terra d’Otranto” PDO [10]. Rossanese and Carolea are well studied and characterized cultivars diffused in the Calabria Region [28,29]. This 126 monocultivar samples database was used to build a partial least squares discriminant analysis (PLS-DA) model by which, a total of 241 Coratina based commercial blends samples obtained using selected cultivars from specific geographical origins produced over four consecutive harvesting years were classified by prediction. It should be underlined that our assignment purpose was limited to assess, with statistical models, the samples compliance with the expected blend characteristics defined by Coratina and a range of sweeteners monocultivar reference oils from specific Italian geographical origins. On the other hand, the comparison of the considered blends with other extra virgin olive oils originating from other countries and the same or other cultivars, clearly requires the use of different models and reference samples. In addition to the compliance with the Coratina based blends, defined by the reference oils, we have also studied a possible blend classification according to their expected bitterness and/or pungency characteristics. This further ranking was evaluated considering, for the studied blend samples, their classification scores for the Coratina with respect to sweeteners reference oils. The analysis of the discrimination between Coratina and sweetener cultivars was then refined by performing pairwise OPLS-DA and considering sweetener cultivars as a single class. Two different OPLS-DA models were therefore built using both the standard zg NMR spectra and the combined zg noesy NMR spectra. These latter better accounts for the phenolic components responsible of the blends bitterness/pungency characteristics essentially due to their expected Coratina content. The studied blend samples were classified by prediction on these two models and the correlation observed for the resulting classification scores suggests a reasonable efficiency of both models. This NMR-bases method could be profitably used as a tool to classify commercial oil samples, putting a gate around high quality blend EVOOs and defining their characteristics with respect to the blend constituents.

## 2. Materials and Methods

Chemicals: All chemicals for analysis were of analytical grade: CDCl_3_ (99.8 atom %D) and tetramethylsilane, TMS (0.03 *v*/*v*%) were purchased from Armar Chemicals (Döttingen, Switzerland).

### 2.1. Sampling

A total of 126 monocultivar samples collected from different selected areas of southern Italian regions (Coratina 74, Cima di Mola 15, Ogliarola Barese 10, Cellina di Nardò 14 from Apulia; Rossanese 3 and Carolea 10 from Calabria) and 241 of 100% Italian commercial blend EVOO samples produced over four consecutive harvesting years (2015/2016; 2016/2017; 2018/2019; 2019/2020) were provided by Certified Origins Italia s.r.l. The different size of the reference samples number was chosen according to their provider declared use in the blends production. Being the model designed for Coratina based blend classification, the number of Coratina oils samples was higher in order to take into account the prevalence of Coratina cultivar and its variability within Apulia Region [27]. All the samples were stored in sealed dark glass bottles at room temperature in the dark until NMR analysis. The information reported on the label of each sample was described in detail in Table 1.

### 2.2. Sample Preparation for NMR Analyses

Each sample was prepared by dissolving ~140 mg of olive oils in a volume of deuterated chloroform (CDCl_3_), calculated on the basis of the ratio of 13.5% oil/86.5% CDCl_3_, *w*/*w* (standard Bruker methodology). Then, 600 μL of the prepared mixture was transferred into a 5 mm diameter NMR tube and subjected to spectroscopic analysis. Oil samples were provided by the producers before bottling. According to the previously published work flow chart illustrating the procedures sequence for blend EVOO origin assessment [12] ^1^H NMR analyses were performed within one month of receiving the samples, within the optimal shelf life [30].

### 2.3. Acquisition and Processing of ^1^H NMR Spectra

^1^H NMR spectra were acquired using a Bruker Avance spectrometer (Bruker Italia, Milano, Italy) operating at proton frequency of 400.13 MHz, T = 300 K, equipped with a PABBI 5-mm inverse detection probe with a z axis gradient coil. The experiments were conducted under full automation, after loading individual samples on a Bruker Automatic Sample Changer (BACS-60), interfaced with the IconNMR software (Bruker Italia, Milano, Italy). In order to characterize fatty acids signals and to enhance signals of the minor components by the suppression of these strong fatty acids signals respectively, for each sample, both a standard ^1^H zg NMR and a multi-suppressed ^1^H noesygpps NMR (with suppression of strong fatty acids signals) experiments were performed. Each ^1^H NMR spectrum was acquired following the conditions: zg pulse program, 64 k time domain, spectral amplitude 20.5555 (8223.685 Hz), p1 12.63 μs, pl1 −1.00 db, 16 repetitions. Each multi-suppressed spectrum was acquired under the conditions: noesygpps1d.comp2 pulse program, 32 k time domain, spectral amplitude 20.5555 (8223.685 Hz), p1 12.63 μs, pl1 −1.00 db, 32 repetitions. The chemical shifts of sample signals were calculated according to the internal standard (TMS), whose signal was set at 0 ppm. The spectra were acquired and processed using the Topspin 3.1 software (Bruker Italia, Milano, Italy). ^1^H NMR spectra were segmented in rectangular fixed (0.04 ppm width) buckets and integrated by Amix 3.9.15 (Analysis of Mixture, Bruker BioSpin GmbH, Rheinstetten, Germany) software. Bucketing of ^1^H zg and ^1^H noesygpps NMR spectra were performed within the range 10.0–0.5 ppm and 10.0–5.6 ppm, respectively. In both cases, the spectral region between 7.6 and 6.9 ppm was excluded to eliminate from the analysis the residual solvent signal peaks area. In order to minimize small differences due to olive oil concentration and/or experimental conditions among samples the total sum normalization was then applied [31]. The Pareto scaling procedure, performed by dividing the mean-centered data by the square root of the standard deviation, was applied to the variables. The data tables obtained with all the aligned buckets row reduced spectra were used for statistical analysis.

### 2.4. Multivariate Statistical Analysis Applied to NMR Spectroscopy Data

After the data processing, multivariate statistical analysis, was then performed by using the Simca-P version 14 (Sartorius Stedim Biotech, Umeå, Sweden) software. In particular, unsupervised principal components analysis (PCA) and supervised partial least squares discriminant analysis (PLS-DA) and orthogonal partial least squares discriminant analysis (OPLS-DA) were performed. PCA is a chemometric method applied in order to extract the maximum information from a multivariate data structure, reducing it in a few linear combinations of the variables [32]. The PCA is used, in the first data processing step, to obtain a general overview of the samples distribution and their possible grouping in homogeneous clusters also identifying the presence of possible outliers [33]. The correlation between the clusters distribution of the analyzed samples and the considered classes (such as variety and/or geographical origin), is therefore assessed by supervised multivariate statistical analyses. PLS-DA is the most used supervised analysis for the discrimination between samples classes with different characteristics [34]. The PLS-DA is performed in order to obtain the maximum separation between groups of observations and information about the variables responsible for the observed separation, by rotating the main components (the axes that express the variance of the data) [35]. OPLS-DA is a modification of the PLS-DA technique which filters out variation not directly related to the discriminating response. This is realized by separating the portion of the variance useful for predictive purposes from the non-predictive variance (which is made orthogonal). The result is a model characterized by an improved interpretability [36]. In our study when we considered six categories (all the different cultivars) PLS-DA rather than OPLS-DA analysis was preferred for further classification purposes [37]. On the other hand, OPLS-DA analysis was used in the pairwise comparisons between Coratina and Sweetener cultivars (considered as a single class) in order to better specify the molecular components responsible for the observed discrimination, being a superior discriminating tool for two classes models [37]. The robustness of the predictive ability for the OPLS/PLS-DA model was evaluated by the misclassification tables and classification list. Classification list provides the overall classification results, according to the predicted values for Y variables (YPredPS) of observations in the prediction set. Membership of a class depends upon matching the value of YPredPS (classification score). A value close to one indicates membership of the workset class. Class membership was defined as follows; YPredPS < 0.35 (also negative values); observation does not belong to the class; 0.35 < YPredPS < 0.65; observation is borderline; YPredPS > 0.65; observation belongs to the class [38]. The misclassification table is complementary to the classification list but presents the classification results in a more compact format. The internal cross-validation default method (7-fold) and the permutation test (40 permutations), both available on the SIMCA-P software [38,39], were used in order to validate the statistical models. The quality of the obtained models was described by the R^2^ and Q^2^ parameters. The first (R^2^) is a cross validation parameter indicating the portion of data variance explained by the models and represents the goodness of fit. R^2^X and R^2^Y indicate the fraction of variance of the X and Y matrix, respectively. The second (Q^2^) is a goodness-of-prediction parameter representing the portion of variance in the data predictable by the model. Values of R^2^X(cum), R^2^Y(cum) and Q^2^(cum) represent cumulative R^2^X, R^2^Y and Q^2^ up to the specified component. The minimal number of necessary components can be defined since R^2^(cum) and Q^2^(cum) parameters show completely diverging behavior as the model complexity increases [39]. For each of these three model parameters a value greater than 0.5 indicates good model quality [25]. The variables responsible for the observed discrimination (loadings) were identified by using the statistical tool S-line plot. The S-line plot, which creates a plot of the loading vectors for two components (usually the first two) is specifically tailor-made for NMR spectroscopy data. Indeed, this plot resembles a NMR spectrum where the original buckets representing the loading and colored according to the absolute value of the correlation p(corr)[1] are displaced in opposite direction depending on their values and the considered components.

## 3. Results and Discussion

### 3.1. New Reference Model: PLS-DA Analysis

**Figure 1:**
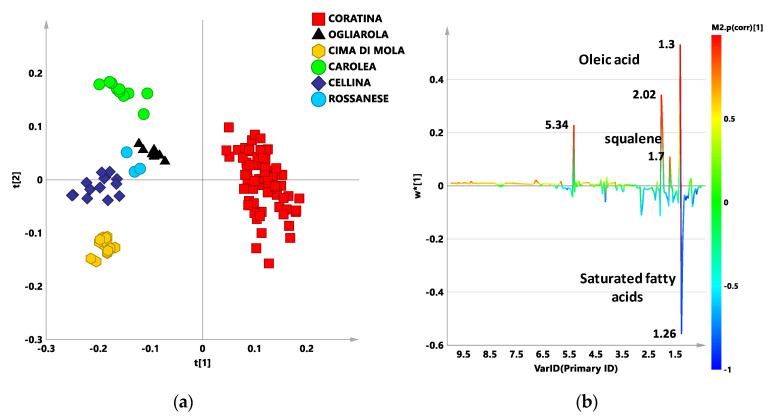
(**a**) Partial least squares discriminant analysis (PLS-DA) t[1]/t[2] scores plot for the monocultivar extra virgin olive oils (EVOOs) data sets. 6 components, R^2^X = 0.953; R^2^Y = 0.750; Q^2^ = 0.711. (**b**) Line plot for the model, indicating the ^1^H Nuclear Magnetic Resonance chemical shifts of the signals, characteristic of specific metabolites, discriminating the classes along t[1] and colored according to the correlation-scaled loading (* p(corr) ≥ |0.5|). w*c[1] axis represented the weighted correlation vector.

Starting from a previously published work [12] we used the same methodology: a reference model built with monocultivar EVOOs samples from specific geographical origins was used to obtain an indication of the composition of a test set Coratina based blends expected to contain the same cultivars from the same geographical areas. Thus, we built a new reference model with 126 reference oils mostly used to produce commercial EVOOs. These were represented by monocultivar EVOO samples from specific geographical origins (Coratina, Cima di Mola, Ogliarola, and Cellina from Apulia; Carolea and Rossanese from Calabria). The supervised PLS-DA analysis performed on the zg NMR spectra (Figure S1) of this new training set gave a model, with a clear differentiation of samples according to cultivar classes as observed in the bi-dimensional t[1]/t[2] scores plot (Figure 1).

In detail, the Coratina samples class was observed clearly separated from the other cultivars, at positive values [0.1 and 0.3] essentially along the t[1] component. Samples of the others cultivars (used as Coratina sweeteners) resulted placed at the negative values [−0.1 and −0.3] of the t[1] component. Moreover, a good separation among the sweetener cultivar samples was observed along the t[2] component. The model resulted stable and with good fit and prediction parameters: (6 components explained 95.3% and 75% of the total variance of the X and Y matrix respectively and 71.1% of predicted variance (R^2^X = 0.953; R^2^Y = 0.750; Q^2^ = 0.711)). PLS-DA model was validated testing for non-casualty by the permutation test performed with 40 cycles of Y variables random permutations for the considered model [34]. The model is considered successfully validated when the R^2^-intercept and Q^2^-intercept do not overcome 0.3–0.4 and 0.05, respectively [38]. The permutation test exhibited Y-intercept and Q^2^-intercept pair values at 0.0163 (R^2^) and –0.307 (Q^2^), 0.0259 (R^2^) and –0.212 (Q^2^), 0.0236 (R^2^) and –0.237 (Q^2^), 0.066 (R^2^) and −0.13 (Q^2^), and 0.0119 (R^2^) and –0.238 (Q^2^) for Coratina, Ogliarola, Cima di Mola, Rossanese, Cellina di Nardò, respectively, thereby demonstrating that the PLS-DA classification model was successfully validated (Figure S2). NMR signals indicating the molecular constituents distinctive for each extra virgin olive oil class and discriminating along the t[1] component were defined by examining the loading line plot for the model (Figure 1b). The variables, indicating signals with chemical shift (δ_H_) at 1.3, 2.02, and 5.34 ppm, corresponding to the methylene (*n*-CH_2_), allylic (-CH_2_CH=CH-) and olefinic (-CH=CH-) protons respectively of oleic acid are higher for Coratina oil with respect to the sweetener cultivars according to literature data [40]. As known, oleic acid (C18:1) is the main monounsaturated fatty acid (MUFA) in olive oil, and it is known to play a protective effect against several diseases, such as liver dysfunction and gut inflammation [41]. Interestingly, signal ascribable to squalene (1.7 ppm) was observed in the loading line revealing higher relative content for Coratina class samples of this triterpene known for important health properties, also able to improve the olive oil stability, and thus shelf life [42,43]. On the contrary, resonance at δ_H_ 1.26 ppm corresponding to the methylene protons (*n*-CH_2_) of the saturated acyl groups showed higher values for the sweetener cultivars than for the Coratina class.

**Figure 2:**
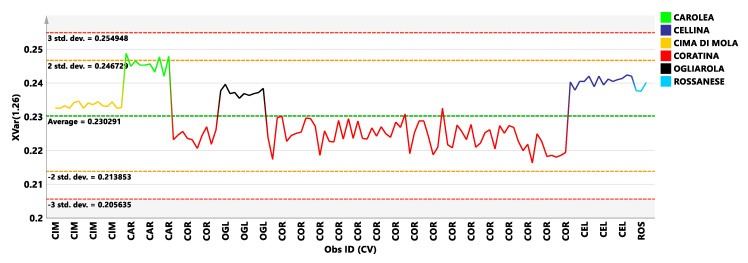
Variable trend plot with control limits of selected discriminating X variable (1.26 ppm). (CIM: Cima di Mola, Car: Carolea; Ogl: Ogliarola; COR: Coratina; CEL: Cellina di Nardò, ROS: Rossanese).

The analysis of the variable trend plot for the discriminating 1.26 ppm variable (Figure 2), showed a significant higher content of saturated fatty acid, associated with sweetness flavour characteristic of the Carolea samples class. The discrimination among the sweetener cultivars, from the analysis of the 1.26 ppm bucket trend plot. Carolea cultivar is known to be characterized by high level of palmitic (C16) and stearic acids [28].

**Figure 3:**
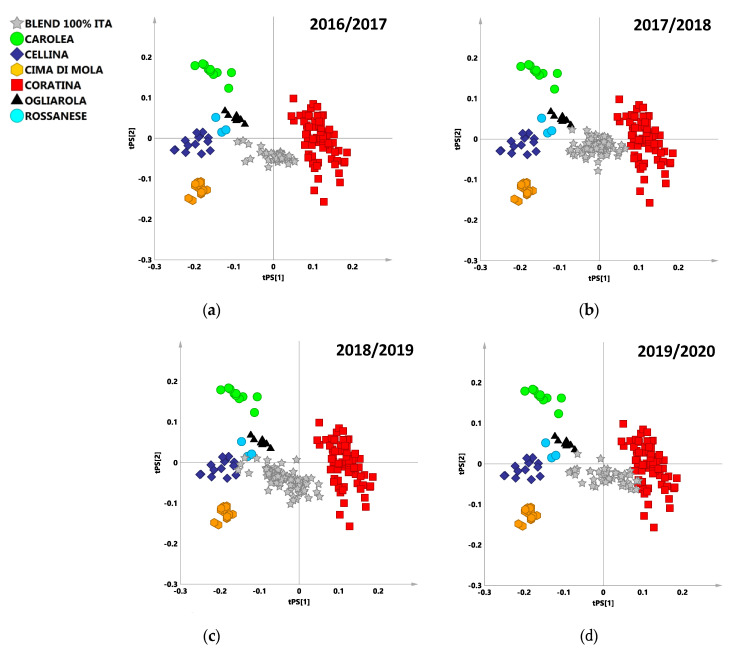
PLS-DA predicted scores plot for the reference model data sets. Prediction set samples, from 2016/2017 (**a**) 2017/2018 (**b**), 2018/2019 (**c**), and 2019/2020 (**d**) were indicated as grey five points star. 6 components gave R^2^X = 0.953; R^2^Y = 0.750; Q^2^ = 0.711.

The obtained model was used to perform the ^1^H NMR analysis of commercial blend samples from four different harvesting years (Experimental section). All the samples were declared as Coratina based blends containing also Ogliarola, Cellina, Cima di Mola, Rossanese, and Carolea as “sweeteners” cultivars. In order to analyze the resulting classification scores with respect to the reference classes, each prediction set, constituted by the Italian commercial blends for a specific harvesting year, was predicted into the PLS-DA model (Figure 3a–d). Each blend sample was classified according to its classification score which reflects the blend content (Table 2). Classification scores above a fixed value limit of 0.65 resulted in a sample assignment to that specific class. In all the other case, the samples were ranked as borderline between the classes resulting with a classification score below 0.65 for that sample. On the other hand, all the analyzed oil test sets resulted essentially mixed composition blends based on Coratina (with smoother cultivars) according to their classification score for Coratina higher than 0.35 (Table S1). The PLS-DA class discrimination occurs according to the differences in the spectral fingerprints for sweeteners with respect to Coratina cultivar essentially observed along t[1] component and already discussed (Figure 1b). Moreover the discriminating ability of the PLS-DA model also results from the differences in the spectral fingerprints among the sweetener cultivars observed along t[2] component (Figure S3). Specific information could be obtained also by examining the line plot for the model, indicating the ^1^H NMR chemical shifts of the signals, characteristic of specific metabolites, discriminating the classes along t[2]. Higher relative content of saturated fatty acids (1.26 ppm corresponding to the methylene of the saturated acyl group) were observed for Carolea cultivar, whereas higher level of polyunsaturated acyl groups (PUFA), (signals at 1.34, 2.38, 2.78 ppm) were observed for Cima di Mola cultivar (Figure S3).

#### 3.1.1. 2016/2017 Harvesting Year

A total of 38 100% Italian commercial samples were classified by prediction in the PLS-DA model (Figure 3a). From the predicted scores plot the commercial blends confirmed their consistency with cultivars from the specific geographical origins declared by the supplier. Predicted samples of mixed cultivars oils set were clustered in the middle of the scores plot slightly closer to the Coratina class, except for a small subset of samples clearly placed close to the “sweeteners” cultivar. The analysis of the classification scores reported in the Classification list (Table S1) and summarized in the misclassification table (Table 2), revealed as most of the predicted samples (33) were assigned to Coratina class (classification scores for Coratina > 0.65). One of the samples resulted assigned to Carolea class, and four of the total were not assigned to any specific class. Nevertheless, for all the samples assigned to cultivars different from Coratina or not assigned to a specific cultivar a classification score for Coratina higher than 0.35 was in any case observed (Table S1).

#### 3.1.2. 2017/2018 Harvesting Year

A total of 74 100% Italian commercial samples were classified by prediction in the PLS-DA model (Figure 3b). The bi-dimensional plot for the model revealed a clear compact clustering of the commercial samples in the middle of the predicted scores plot. From the predicted scores plot the placement of the commercial blends confirmed the consistency with cultivars from the specific geographical origin declared by the supplier. From the classification score analysis, the prediction of the commercial samples was evaluated. Most of the samples (63) were assigned to Coratina class (classification scores for Coratina > 0.65) while, 11 samples were not assigned to any specific class, being the predicted values borderline between classes and below the fixed 0.65 limit (Table 2, Table S1). Moreover, in this case, for most of the samples assigned to cultivars different from Coratina or not assigned to a specific cultivar, a classification score for Coratina higher than 0.35 was observed. Only three samples were characterized by a relatively smaller classification score for Coratina (0.20 < 0.35) (Table S1).

#### 3.1.3. 2018/2019 Harvesting Year

A total of 80 100% Italian commercial samples were classified by prediction in the PLS-DA model. As observed in the bi-dimensional predicted scores plot for the model (Figure 3c) the predicted set is consistent with cultivars from the specific geographical origin declared by the supplier. In particular, predicted samples of mixed cultivars oil set were clustered in the middle of the scores plot confirming their blended composition. The misclassification table reported the assignment according to the prediction classification scores (Table 2). A relevant number of the test samples (24) were directly assigned to Coratina class, confirming that the Coratina is the predominant cultivar in the provided blend samples. A total of 20 samples were also assigned to Cellina class, also accordingly to their position in the predicted scores plot. Some samples (17) overlapping between Cellina–Coratina classes were assigned to a combined Cellina Coratina class. A further subset of test samples (19) was not assigned to any specific class, being their predicted classification score values borderline between classes and below the fixed 0.65 limit (Table S1). Anyhow, for all the samples assigned to cultivars different from Coratina or not assigned to a specific cultivar a classification score for Coratina higher than 0.35 was observed (Table S1).

#### 3.1.4. 2019/2020 Harvesting Year

A total of 49 100% Italian commercial samples were classified by prediction in the PLS-DA model. As observed in the bidimensional scores plot for the model (Figure 3d) the predicted set is consistent with cultivars from the specific geographical origin declared by the supplier. In this harvesting year, the predicted test sample which constitute the central cluster of the scoreplot appears somehow more evenly distributed along the first component tPS[1]. The misclassification table (Table 2) confirmed the visual inspection of the scores plot. A total of 39 samples were directly classified as Coratina or combined Cellina Coratina class, nine samples were assigned to Cellina class and one blend was not assigned to any specific class. Again, all the samples not directly assigned to Coratina or combined Coratina classes were in any case characterized by a classification score for Coratina higher than 0.35 (Table S1).

As known, blended EVOOs are produced by combining monocultivar oils with different flavor and taste profiles in order to meet the demands of national and international market. In this study a 126 monocultivar samples database was used to build a PLS-DA model. A total of 241 Coratina based commercial blends samples obtained using selected cultivars from specific geographical origins produced over four consecutive harvesting years were classified by prediction using the PLS-DA model. The classification scores obtained for the test samples of the considered harvesting years reflected their expected blend composition and could provide to the producers useful information on the organoleptic aspects of the product (e.g., bitter and spicy). As already reported a minimal harvesting year effect is expected for the Coratina based blends [13]. Therefore, the observed slight variability of the blend samples according to the production year could be possibly ascribed to a variation in the EVOOs suppliers. Thus, the here reported tailor-made dataset could be used to assess the quality of the commercial blend samples with similar declared composition and highlight possible deviations with respect to the expected standard product features. Our assignment purpose was limited to assess, with statistical models, the samples compliance with the expected blend characteristics defined by Coratina and a range of sweeteners monocultivar reference oils from specific geographical origins. This assignment was simply performed according to the provider declaration of the used cultivars, without considering the percentage composition. On the other hand, a multivariate analysis (MVA)-based NMR approach—such as PLS regression (PLSR)—proved a very useful tool to assess quantitative blend composition although limited to simplified binary system [24].

### 3.2. Coratina vs. Sweetners Cultivars: OPLS-DA Pairwise Analysis

**Figure 4:**
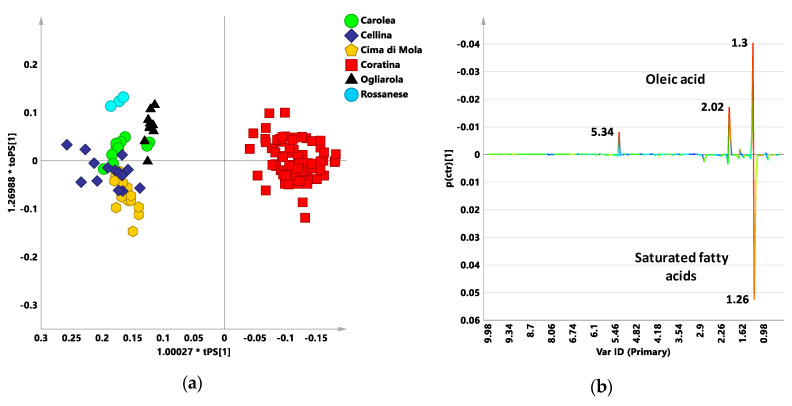
(**a**) Orthogonal partial least squares discriminant analysis (OPLS-DA) t[1]/t[2] scores plot for the monocultivar EVOOs data sets built with the zg NMR spectra. One predictive and one orthogonal components account for R^2^X = 0.673; R^2^Y = 0.951; Q^2^ = 0.945. 1.0027*tPS[1]; 1.26988*toPS[1]: axes scaled proportionally to R^2^X. (**b**) S-line for the model visualizing the p(ctr)[1] loading colored according to the absolute value of the correlation loading, p(corr)[1].

In order to obtain a possible classification of the examined blends according to their expected bitterness and/or pungency characteristics, a pairwise OPLS-DA analysis was further performed by considering Coratina cultivar class and the sweeteners cultivars grouped together as a single class. In this respect the resulting OPLS-DA models could be considered as a simplified classification tools with respect to the PLS-DA models described above. In the first instance the default bucket table (zg acquisition) was used for this purpose. As expected, the obtained model was characterized by a marked separation between the two class and described by excellent descriptive and predictive parameters: one orthogonal and one predictive components gave R^2^X = 0.673; R^2^Y = 0.951; Q^2^ = 0.945 (Figure 4a). By examining the S line plot of the loadings for the model (Figure 4b) the molecular components discriminant for the two classes were defined. As already described for the signals, discriminating the classes along t[1] in the zg NMR PLS-DA model, the variables, indicating signals with chemical shift (δ_H_) at 1.3, 2.02, and 5.34 ppm, ascribable to oleic acid are higher for Coratina oil. Moreover, resonance at δ_H_ 1.26 ppm corresponding to the saturated acyl groups showed higher values for the sweetener cultivars than for the Coratina class.

**Figure 5:**
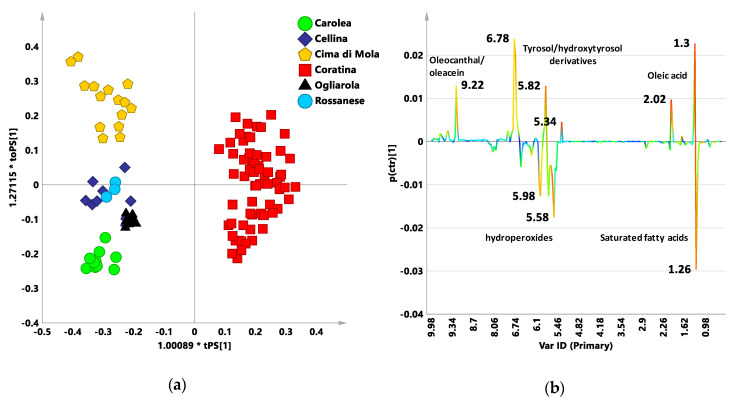
(**a**) OPLS-DA t[1]/t[2] scores plot for the monocultivar EVOOs data sets built with bucket reduced combined zg-noesy NMR spectra. One predictive and one orthogonal components account for R^2^X 0.579; R^2^Y 0.945; Q^2^ 0.936; 1.0089*tPS[1]; 1.27115*toPS[1]: axes scaled proportionally to R^2^X. (**b**) S-line plot for the model visualizing the p(ctr)[1] loading colored according to the absolute value of the correlation loading, p(corr)[1].

Moreover, because the phenolic compounds characteristic of the Coratina cultivar are responsible for the bitterness and/or pungency sensory attributes of the Italian EVOO blends, a further refined model was also considered. As known, the organoleptic attributes of pungency and bitterness in olive oil are attributed to phenolic compounds [44,45]. Furthermore, phenolic compounds are also the basis of oxidative stability and of the main nutritional properties of oils and this makes the analysis of the contribution of polyphenols essential for the extra virgin olive oil research [44]. In order to enhance the polyphenol contribution, another pairwise OPLS-DA analysis, using combined bucket reduced NMR spectra was performed. The combined bucket table was generated by combining in one matrix the ^1^H noesygpps NMR spectra (Figure S4) (within the range 10.0–5.6 ppm) and the ^1^H zg NMR spectra (within the range 5.6–0.5 ppm) as previously reported [10]. Further in this case, the obtained OPLS-DA model for the two classes (Coratina and sweeteners cultivars) showed a clear separation between the groups and very good statistical parameters (one predictive and one orthogonal component, R^2^X = 0.579; R^2^Y = 0.945; Q^2^ = 0.936) (Figure 5a). The S line plot of the loadings for the model showed the molecular components of triglycerides and unsaponifiable fractions as discriminant for the two groups (Figure 5b). Higher oleic acid could be observed for Coratina (1.3, 2.02, and 5.34 ppm), as already found in the t[1] line and S line plots of the loadings for the PLS-DA (Figure 1b) and OPLS-DA (Figure 4b) models built with the standard zg NMR spectra. Moreover, Coratina class was also characterized by higher content of phenolic moieties of oleuropein and ligstroside aglycones such as tyrosol and hydroxytyrosol derivatives (6.78 ppm), as well as for higher secoiridois derivatives oleocanthal and oleacein (9.22 ppm). These secoiridoic phenolics are known for their antioxidant and anti-inflammatory properties and organoleptic association with bitterness and pungency [45]. Therefore, these signals could be also related to a possible classification of the examined Coratina based blends according to their expected bitterness and/or pungency characteristics. On the other hand, only a specific correlation study with organoleptic analyses could buttress this features assignment to the oils classified by the model. In the case of the sweeteners class, again, the variables (buckets) ascribable to saturated fatty acids (1.26 ppm) were observed as discriminating together with signals of compounds associated to degradation processes (5.98, 5.58 ppm) [46].

The predictive capability of the two models described by the high Q^2^ values was then tested by classifying the whole Italian 100% EVOOs blend test set (Table 3 and Table S2).

For the OPLS-DA model built with the zg NMR spectra, the classification scores analysis revealed as more of the 98% out of the considered 233 observations showed classification scores values for Coratina higher than 0.35. In particular, 56% and 43% showed classification scores for Coratina above 0.65 and between 0.35 and 0.65, respectively. Only 1.2% of the classification scores showed values lower than 0.35 (although above 0.25) with the predicted samples assigned to Sweeteners class. A slightly lower percentage (96%) of the considered 233 observations showed classification scores values for Coratina higher than 0.35 predicted on the OPLS-DA model built with bucket reduced combined zg-noesy NMR spectra. Among them, 34% and 62% showed classification scores for Coratina above 0.65 and between 0.35–0.65, respectively. Furthermore, in this case, a small number of the predicted samples (4%) were assigned to sweeteners cultivars, showing a classification scores values for Coratina lower than 0.35 (although above 0.26). The model built with the combined bucket table, seems to be characterized by a greater selectivity with respect to prediction scores values for Coratina when compared to the “standard” major components model (zg spectra bucket table). Indeed, the combined buckets model, better accounts for the presence of specific components such as polyphenols and other molecules related to the expected bitterness and/or pungency characteristics of Coratina based blend oils. Therefore, the use of the combined buckets model, restricting the requirements associated to the predictions scores for Coratina, results in an increased percentage of samples not assigned to any specific class or assigned to the sweeteners class (classification scores values for Coratina between 0.35 and 0.65 or below 0.35, respectively). The relation between the classification scores for the two models (Figure S5) was also investigated. The regression was significant with acceptable R^2^ value (0.7029) and slope (0.86) revealing a good correlation between the predictive OPLS-DA models built with zg and combined zg-noesy bucket reduced spectra. Therefore, both models could be profitably used to generally classify the studied Coratina based blends, from specific geographical origins, taking into account only the major lipid fraction (standard zg spectra) or also the minor phenolic component (combined zg-noesy spectra). This classification may also constitute an indirect method to rank commercial Coratina based blend samples according to their expected bitterness and/or pungency characteristics.

## 4. Conclusions

In this work, based on ^1^H NMR data, a total of 241 of commercial 100% Italian blend olive oil samples from four different harvesting years, were classified by prediction using a reference olive oils database built with 126 monocultivar EVOOs. This dataset includes Carolea, Cellina, Cima di Mola, Coratina, Ogliarola, and Rossanese monocultivar olive oil samples, from specific geographical origins. In particular, a supervised PLS-DA model, was built and used for classification purposes of the commercial blend samples. All the classified commercial blend samples resulted essentially mixed composition blend based on Coratina (with smoother cultivars) according to their resulting classification score for Coratina higher than 0.35.

In order to obtain a simple classification tool for ranking the examined blends according to their expected bitterness and/or pungency characteristics, an OPLS-DA analysis was also used in a pairwise comparisons between Coratina and Sweetener cultivars considered as a single class. For this purpose, two different OPLS-DA models were obtained by using both the default (zg acquisition) and the combined (zg 0.5–5.6 and noesygpps 5.6–10.0 ppm) bucket reduced ^1^H-NMR spectra datasets. The predictive capability of the two models, both characterized by good Q^2^ values, was tested by evaluating the classification scores for Coratina of the Italian 100% EVOOs blend test set. The analysis revealed as most of the considered 233 observations (98% and 96% for the OPLS-DA models obtained using the default and the combined bucket table, respectively) showed a classification scores values for Coratina higher than 0.35. The combined buckets model, better accounts for the presence of polyphenols and other molecules related to the organoleptic characteristics of Coratina based oils. Accordingly, its higher discrimination power results in an increased percentage of samples (4%) characterized by classification scores values for Coratina below 0.35. Nevertheless, the correlation analysis for the obtained classification scores, showed that both OPLS-DA models could be profitably used to generally classify the studied Coratina based blends, from specific geographical origins, taking into account only the major lipid fraction (standard zg spectra) or also the minor phenolic component (combined zg-noesy spectra). Therefore, tailor-made databases based on ^1^H NMR data of monocultivar oils from specific geographical origins could be profitably used to build a gate around high quality blend EVOOs and define their characteristics with respect to a specific monocultivar reference oils dataset. The described models may also offer an indirect method to classify commercial samples according to their expected bitterness and/or pungency characteristics, although a further specific correlation study with organoleptic analysis is required to buttress this result.

## Figures and Tables

**Table 1:** List of analyzed monocultivar and blend samples.

	Number of Samples	Cultivar	Origin	Harvesting Period
Monocultivar	74	Coratina	Apulia (Italy)	2012/2013; 2013/2014
	10	Ogliarola Barese	Apulia (Italy)	2012/2013; 2013/2014
	15	Cima di Mola	Apulia (Italy)	2013/2014
	10	Carolea	Calabria (Italy)	2012/2013; 2015/2016
	14	Cellina di Nardò	Apulia (Italy)	2012/2013
	3	Rossanese	Calabria (Italy)	2012/2013
100% Italian BLEND	38	Ogliarola Barese + Coratina + Carolea + Cima di Mola + Rossanese + Cellina di Nardò	Apulia (Italy); Calabria (Italy)	2016/2017
	74	Ogliarola Barese + Coratina + Carolea + Cima di Mola + Rossanese + Cellina di Nardò	Apulia (Italy); Calabria (Italy)	2017/2018
	80	Ogliarola Barese + Coratina + Carolea + Cima di Mola + Rossanese + Cellina di Nardò	Apulia (Italy); Calabria (Italy)	2018/2019
	49	Ogliarola Barese + Coratina + Carolea + Cima di Mola + Rossanese + Cellina di Nardò	Apulia (Italy); Calabria (Italy)	2019/2020

**Table 2:** Misclassification table summarizing the classified observations in the 2016/17, 2017/18, 2018/2019, and 2019/2020 prediction sets.

		N° of Samples	Coratina	Ogliarola	Cima di Mola	Carolea	Cellina	Rossanese	Coratina & Cellina	No Class (Ypred ≤ 0.65) ^1^
training set	Coratina	74	74							
	Ogliarola	10	0	10						
	Cima di Mola	15			14					1
	Carolea	10				10				
	Cellina	14								
	Rossanese	3								3
prediction set	2016/2017	38	33		1					4
	2017/2018	74	63							11
	2018/2019	80	24				20		17	19
	2019/2020	49	1				9		38	1

^1^ “0.65” is the fixed value to assign observations to all classes above the limit.

**Table 3:** Misclassification table summarizing the classified observations in the OPLS-DA models built with bucket reduced zg and zg-noesy NMR spectra.

NMR Experiment			N° of Samples	Coratina	Sweeteners	No Class (Ypred ≤ 0.65) ^1^
^1^H zg	training set	Coratina	74	74		
		Sweeteners	52	0	52	
	prediction set	100% Italian EVOO blends	233	130	3	100
^1^H noesygpps	training set	Coratina	74	74		
		Sweeteners	52	0	52	
	prediction set	100% Italian EVOO blends	233	80	9	144

^1^ “0.65” is the fixed value to assign observations to all classes above the limit.

## Supplementary Materials

The following are available online at https://www.mdpi.com/2304-8158/9/12/1797/s1, Table S1: Classification list for the observations (commercial 100% Italian EVOO blends from 2016/2017, 2017/2018, 2018/2019, 2019/2020 harvesting years) predicted on the PLS-DA reference model; Table S2: Classification list for the observations (commercial 100% Italian EVOO blends from 2016/2017, 2017/2018, 2018/2019, 2019/2020 harvesting years) predicted on the OPLS-DA models built with zg and zg combined noesy bucket reduced spectra. Figure S1 Representative zg ^1^H NMR spectra of EVOO sample. Main metabolites are indicated; Figure S2. Permutation test performed with 40 cycles of random permutation of Y variables on PLS-DA models for Coratina, Ogliarola, Cima di Mola, Cellina, Carolea and Rossanese cultivars; Figure S3. Line plot for the Figure 1a model, indicating the ^1^H NMR chemical shifts of the signals, characteristic of specific metabolites, discriminating the classes along t[2] and colored according to the correlation-scaled loading (* p(corr) ≥ |0.5|). w*c[1] axis represented the weighted correlation vector; Figure S4 Representative noesygpps ^1^H NMR spectra of EVOO sample. Main metabolites are indicated; Figure S5. Relationship between classification scores of the commercial blend samples predicted on the bucket reduced combined zg-noesy NMR spectra.

## References

[1] Del Monaco G., Officioso A., D’Angelo S., La Cara F., Ionata E., Marcolongo L., Squillaci G., Maurelli L., Morana A. (2015). Characterization of extra virgin olive oils produced with typical Italian varieties by their phenolic profile. Food Chem..

[2] Jiang H., Chen Q. (2019). Determination of adulteration content in extra virgin olive oil using FT-NIR spectroscopy combined with the BOSS–PLS algorithm. Molecules.

[3] Cicerale S., Lucas L., Keast R. (2012). Antimicrobial, antioxidant and anti-inflammatory phenolic activities in extra virgin olive oil. Curr. Opin. Biotechnol..

[4] ISMEA Istituto di Servizi per il Mercato Agricolo Alimentare. scheda_di_settore_OLIO_aprile_2020. http://www.ismeamercati.it/olio-oliva.

[5] Piarulli L., Savoia M.A., Taranto F., D’Agostino N., Sardaro R., Girone S., Gadaleta S., Fucili V., De Giovanni C., Montemurro C. (2019). A Robust DNA Isolation Protocol from Filtered Commercial Olive Oil for PCR-Based Fingerprinting. Foods.

[6] Consonni R., Cagliani L.R. (2019). NMR Studies on Italian PDO Olive Oils and their Potential in Olive-Tree-Derived Products Characterization. Eur. J. Lipid Sci. Technol..

[7] European Community (2009). Commission Regulation (EC) No 182/2009 of 6 March 2009 amending Regulation (EC) No 1019/2002 on marketing standards for olive oil. Off. J. Eur. Union.

[8] European Community (2012). Commission Implementing Regulation (EU) No 29/2012 of 13 January 2012 on marketing standards for olive oil. Off. J. Eur. Union.

[9] El Riachy M., Bou-Mitri C., Youssef A., Andary R., Skaff W. (2018). Chemical and sensorial characteristics of olive oil produced from the Lebanese olive variety ‘Baladi’. Sustainability.

[10] Del Coco L., De Pascali S.A., Fanizzi F.P. (2014). NMR-metabolomic study on monocultivar and blend salento EVOOs including some from secular olive trees. Food Nutr. Sci..

[11] Del Coco L., De Pascali S.A., Fanizzi F.P. (2014). ^1^H NMR spectroscopy and multivariate analysis of monovarietal EVOOs as a tool for modulating Coratina-based blends. Foods.

[12] Girelli C.R., Del Coco L., Fanizzi F.P. (2016). ^1^H NMR spectroscopy and multivariate analysis as possible tool to assess cultivars, from specific geographical areas, in EVOOs. Eur. J. Lipid Sci. Technol..

[13] Girelli C.R., Del Coco L., Papadia P., De Pascali S.A., Fanizzi F.P. (2016). Harvest year effects on Apulian EVOOs evaluated by ^1^H NMR based metabolomics. PeerJ.

[14] Girelli C.R., Del Coco L., Zelasco S., Salimonti A., Conforti F.L., Biagianti A., Barbini D., Fanizzi F.P. (2018). Traceability of “Tuscan PGI” extra virgin olive oils by ^1^H NMR metabolic profiles collection and analysis. Metabolites.

[15] Mannina L., Sobolev A.P. (2011). High resolution NMR characterization of olive oils in terms of quality, authenticity and geographical origin. Magn. Reson. Chem..

[16] Rongai D., Sabatini N., Del Coco L., Perri E., Del Re P., Simone N., Marchegiani D., Fanizzi F.P. (2017). ^1^H NMR and multivariate analysis for geographic characterization of commercial extra virgin olive oil: A possible correlation with climate data. Foods.

[17] Sacchi R., Mannina L., Fiordiponti P., Barone P., Paolillo L., Patumi M., Segre A. (1998). Characterization of Italian extra virgin olive oils using ^1^H-NMR spectroscopy. J. Agric. Food Chem..

[18] Sacco A., Brescia M.A., Liuzzi V., Reniero F., Guillou G., Ghelli S., van der Meer P. (2000). Characterization of Italian olive oils based on analytical and nuclear magnetic resonance determinations. J. Am. Oil Chem. Soc..

[19] Guillén M.a.D., Ruiz A. (2001). High resolution ^1^H nuclear magnetic resonance in the study of edible oils and fats. Trends Food Sci. Technol..

[20] Sacchi R., Addeo F., Paolillo L. (1997). ^1^H and ^13^C NMR of virgin olive oil. An overview. Magn. Reson. Chem..

[21] Vlahov G. (1999). Application of NMR to the study of olive oils. Prog. Nucl. Magn. Reson. Spectrosc..

[22] Mannina L., Sobolev A.P., Segre A. (2003). Olive oil as seen by NMR and chemometrics. Spectrosc. Eur..

[23] D’Imperio M., Mannina L., Capitani D., Bidet O., Rossi E., Bucarelli F.M., Quaglia G.B., Segre A. (2007). NMR and statistical study of olive oils from Lazio: A geographical, ecological and agronomic characterization. Food Chem..

[24] Girelli C.R., Del Coco L., Fanizzi F.P. (2017). Tunisian extra virgin olive oil traceability in the EEC market: Tunisian/Italian (Coratina) EVOOs blend as a case study. Sustainability.

[25] Ghisoni S., Lucini L., Angilletta F., Rocchetti G., Farinelli D., Tombesi S., Trevisan M. (2019). Discrimination of extra-virgin-olive oils from different cultivars and geographical origins by untargeted metabolomics. Food Res. Int..

[26] Mannina L., D’Imperio M., Capitani D., Rezzi S., Guillou C., Mavromoustakos T., Vilchez M.A.D.M., Fernaández A.H., Thomas F., Aparicio R. (2009). ^1^H NMR-based protocol for the detection of adulterations of refined olive oil with refined hazelnut oil. J. Agric. Food Chem..

[27] Del Coco L., Mondelli D., Mezzapesa G.N., Miano T., De Pascali S.A., Girelli C.R., Fanizzi F.P. (2016). Protected Designation of Origin extra virgin olive oils assessment by nuclear magnetic resonance and multivariate statistical analysis: “Terra di Bari”, an Apulian (Southeast Italy) case study. J. Am. Oil Chem. Soc..

[28] Piscopo A., De Bruno A., Zappia A., Ventre C., Poiana M. (2016). Characterization of monovarietal olive oils obtained from mills of Calabria region (Southern Italy). Food Chem..

[29] Lanteri S., Armanino C., Perri E., Palopoli A. (2002). Study of oils from Calabrian olive cultivars by chemometric methods. Food Chem..

[30] Barbarisi C., Di Stasio M., La Cara F., Nazzaro M., Siano F., Coppola R., Volpe F., De Mattia A., Grazia Volpe M. (2014). Shelf-life of extra virgin olive oils from Southern Italy. Curr. Nutr. Food Sci..

[31] van den Berg R.A., Hoefsloot H.C., Westerhuis J.A., Smilde A.K., van der Werf M.J. (2006). Centering, scaling, and transformations: Improving the biological information content of metabolomics data. BMC Genom..

[32] Jackson J.E. (2005). A User’s Guide to Principal Components.

[33] Kettaneh N., Berglund A., Wold S. (2005). PCA and PLS with very large data sets. Comput. Stat. Data Anal..

[34] Triba M.N., Le Moyec L., Amathieu R., Goossens C., Bouchemal N., Nahon P., Rutledge D.N., Savarin P. (2015). PLS/OPLS models in metabolomics: The impact of permutation of dataset rows on the K-fold cross-validation quality parameters. Mol. BioSyst..

[35] Wold S., Eriksson L., Trygg J., Kettaneh N. (2004). The PLS Method–Partial Least Squares Projections to Latent Structures–and Its Applications in Industrial RDP (Research, Development, and Production).

[36] Trygg J., Wold S. (2002). Orthogonal projections to latent structures (O-PLS). J. Chem..

[37] Boccard J., Rutledge D.N. (2013). A consensus orthogonal partial least squares discriminant analysis (OPLS-DA) strategy for multiblock Omics data fusion. Anal. Chim. Acta.

[38] Eriksson L., Byrne T., Johansson E., Trygg J., Vikström C. (2013). Multi-and Megavariate Data Analysis Basic Principles and Applications.

[39] Wheelock Å.M., Wheelock C.E. (2013). Trials and tribulations of ‘omics data analysis: Assessing quality of SIMCA-based multivariate models using examples from pulmonary medicine. Mol. BioSyst..

[40] Barison A., Pereira da Silva C.W., Campos F.R., Simonelli F., Lenz C.A., Ferreira A.G. (2010). A simple methodology for the determination of fatty acid composition in edible oils through ^1^H NMR spectroscopy. Magn. Reson. Chem..

[41] Cariello M., Contursi A., Gadaleta R.M., Piccinin E., De Santis S., Piglionica M., Spaziante A.F., Sabbà C., Villani G., Moschetta A. (2020). Extra-Virgin Olive Oil from Apulian Cultivars and Intestinal Inflammation. Nutrients.

[42] Ansari A.A., Gill S.S., Abbas Z.K., Naeem M. (2016). Plant Biodiversity: Monitoring, Assessment and Conservation.

[43] Pacetti D., Scortichini S., Boarelli M.C., Fiorini D. (2019). Simple and rapid method to analyse squalene in olive oils and extra virgin olive oils. Food Control.

[44] Favati F., Condelli N., Galgano F., Caruso M.C. (2013). Extra virgin olive oil bitterness evaluation by sensory and chemical analyses. Food Chem..

[45] Demopoulos V., Karkoula E., Magiatis P., Melliou E., Kotsiras A., Mouroutoglou C. Correlation of Oleocanthal and Oleacein Concentration with Pungency and Bitterness in “Koroneiki“ Virgin olive oil. Proceedings of the II International Symposium on Horticulture in Europe 1099.

[46] Ruiz-Aracama A., Goicoechea E., Guillén M.D. (2017). Direct study of minor extra-virgin olive oil components without any sample modification. ^1^H NMR multisupression experiment: A powerful tool. Food Chem..

