# Supplementary material for: 1H NMR Spectroscopy to Characterize Italian Extra Virgin Olive Oil Blends, Using Statistical Models and Databases Based on Monocultivar Reference Oils
Source: Foods. 2020 Dec 3;9(12):1797. doi: 10.3390/foods9121797 (PMC7761774; doi:10.3390/foods9121797)
Supplement: Supplementary file 1 [file foods-09-01797-s001.pdf]

Supplementary

^{1}H NMR spectroscopy to characterize Italian extra virgin olive oil blends, using statistical models and databases based on monocultivar reference oils.

Chiara Roberta Girelli[†1], Francesca Calò[†1], Federica Angilè [1], Lucia Mazzi [b], Daniele Barbini [b] and Francesco Paolo Fanizzi [1,*]

[1] Department of Biological and Environmental Sciences and Technologies, University of Salento, Prov.le Lecce-Monteroni, 73100 Lecce, Italy; chiara.girelli@unisalento.it (C.R.G.); francesca.calo@unisalento.it (F.C.); federica.angile@unisalento.it (F.A.); fp.fanizzi@unisalento.it (F.P.F.)

[2] Certified Origins Italia srl, Località il Madonnino, 58100 Grosseto, Italy; lucia.mazzi@oleificioolma.it (L.M.); daniele.barbini@certifiedorigins.it (D.B.)

[†] These authors contributed equally to this work.

* Correspondence: fp.fanizzi@unisalento.it; Tel.: +39-0832-29265

Received: date; Accepted: date; Published: date

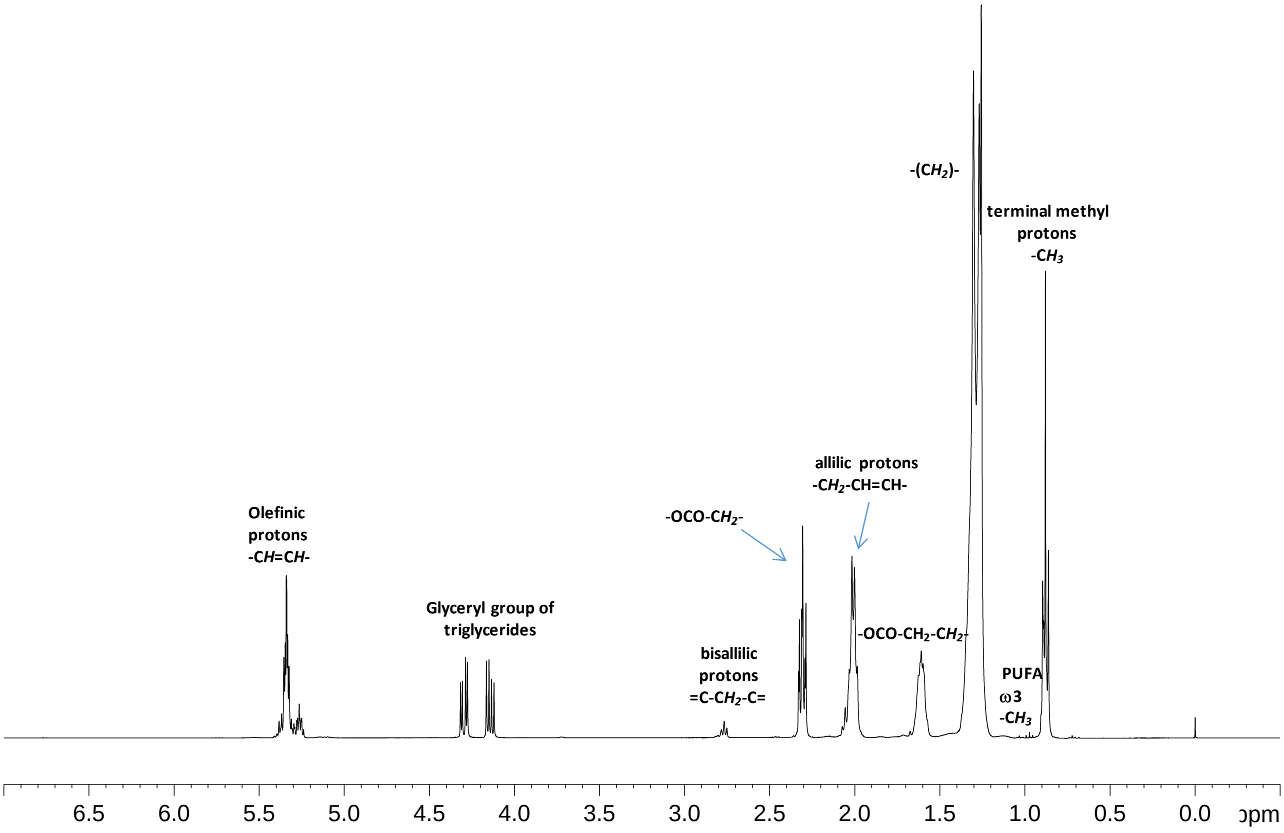

Figure S1 Representative zg ^{1}H NMR spectra of EVOO sample. Main metabolites are indicated

Table S1: Classification list for the observations (commercial 100% Italian EVOO blends from 2016/2017, 2017/2018, 2018/2019, 2019/2020 harvesting years) predicted on the PLS-DA reference model

id	year	M2.YPredPS[6] ($M2.DA (CORATINA))	M2.YPredPS[6]($M 2.DA (OGLIAROLA))	M2.YPredPS[6]($M 2.DA (CIMA DI MOLA))	M2.YPredPS[6]($M 2.DA (CAROLEA))	M2.YPredPS[6]($ M2.DA (CELLINA))	M2.YPredPS[6]($M2.DA (ROSSANESE))
1	2016	0.483306	-0.206717	0.397792	0.0151909	0.416071	-0.105643
2	2016	0.477574	-0.339106	0.592089	0.119924	0.294117	-0.144598
3	2016	0.581582	-0.40064	0.417568	0.0625455	0.467365	-0.12842
4	2016	0.697893	-0.206826	0.447961	-0.157274	0.368563	-0.150318
5	2016	0.6739	-0.292378	0.481212	-0.0921833	0.386663	-0.157213
6	2016	0.694838	-0.333877	0.364306	-0.0761449	0.459921	-0.109043

7	2016	0.860345	-0.38854	0.377237	-0.15303	0.478162	-0.174174
8	2016	0.905531	-0.324501	0.364256	-0.253609	0.488866	-0.180542
9	2016	0.748907	-0.280372	0.544235	-0.177087	0.347236	-0.182919
10	2016	0.857429	-0.376453	0.394853	-0.176192	0.460657	-0.160293
11	2016	0.844742	-0.34615	0.379275	-0.166544	0.438842	-0.150165
12	2016	0.847693	-0.307566	0.422144	-0.203929	0.421895	-0.180236
13	2016	0.733759	-0.254368	0.645243	-0.181982	0.225243	-0.167895
14	2016	0.909626	-0.301466	0.370129	-0.244126	0.431536	-0.1657
15	2016	0.887006	-0.379104	0.369125	-0.193199	0.520488	-0.204316
16	2016	0.816014	-0.391045	0.525661	-0.139542	0.37492	-0.186007
17	2016	0.867792	-0.354268	0.348478	-0.18908	0.510228	-0.183151
18	2016	0.828278	-0.328623	0.449035	-0.209109	0.422756	-0.162336
19	2016	0.739322	-0.334356	0.381951	-0.137418	0.513967	-0.163466
20	2016	0.870085	-0.363029	0.376088	-0.162828	0.452661	-0.172978
21	2016	0.845239	-0.295561	0.292527	-0.205255	0.504914	-0.141864
22	2016	0.920628	-0.31577	0.399427	-0.247509	0.426865	-0.183642
23	2016	0.680975	-0.346482	0.341843	-0.0392437	0.500266	-0.137359
24	2016	0.844802	-0.373897	0.545985	-0.176954	0.371636	-0.211572
25	2016	0.48265	-0.292847	0.590596	0.13566	0.158234	-0.0742927
26	2016	0.76841	-0.198764	0.400698	-0.206874	0.397846	-0.161317
27	2016	0.750522	-0.244725	0.461959	-0.132354	0.321645	-0.157047
28	2016	0.775942	-0.218636	0.527637	-0.166957	0.250825	-0.168811
29	2016	0.78131	-0.269985	0.418625	-0.0854982	0.274838	-0.11929
30	2016	0.911632	-0.342532	0.467918	-0.154251	0.296893	-0.17966
31	2016	0.962996	-0.416344	0.295412	-0.169485	0.467393	-0.139972
32	2016	0.560145	-0.197706	0.654553	-0.0653268	0.197073	-0.148737
33	2016	0.899661	-0.23487	0.320446	-0.247496	0.415056	-0.152797
34	2016	0.79723	-0.310256	0.489613	-0.107101	0.274957	-0.144443
35	2016	0.832091	-0.321924	0.463566	-0.110862	0.291292	-0.154163
36	2016	0.856198	-0.358351	0.462548	-0.0980975	0.29383	-0.156127
37	2016	0.933109	-0.316243	0.378439	-0.164115	0.28273	-0.113921
38	2016	0.884517	-0.258531	0.50403	-0.190961	0.208037	-0.147092
39	2017	0.718086	-0.37971	0.390517	0.00558911	0.39602	-0.130503
40	2017	0.677963	-0.349105	0.420287	-0.0941812	0.498086	-0.15305
41	2017	0.712079	-0.409771	0.321502	0.0191582	0.469024	-0.111993
42	2017	0.623874	-0.395115	0.48008	0.0936679	0.315418	-0.117924
43	2017	0.60561	-0.333135	0.411671	0.0692089	0.369352	-0.122707
44	2017	0.840946	-0.440708	0.260446	0.00203943	0.473013	-0.135736
45	2017	0.757051	-0.374156	0.377817	-0.0446903	0.452873	-0.168895
46	2017	0.837021	-0.471115	0.331625	0.0251498	0.435854	-0.158535
47	2017	0.68525	-0.369275	0.276355	0.0885651	0.443627	-0.124523
48	2017	0.736681	-0.390242	0.443451	-0.000364244	0.286407	-0.0759327
49	2017	0.68574	-0.362656	0.483618	0.0494589	0.316197	-0.172358
50	2017	0.749598	-0.358309	0.33588	0.014332	0.405733	-0.147234
51	2017	0.890821	-0.320155	0.270991	-0.192595	0.554574	-0.203635
52	2017	0.888521	-0.474846	0.211855	0.0128013	0.513366	-0.151697
53	2017	0.763099	-0.349669	0.422725	-0.0611993	0.402656	-0.177611
54	2017	0.720058	-0.040526	0.270149	-0.252619	0.472226	-0.169287
55	2017	0.778673	-0.00877447	0.27474	-0.250638	0.369442	-0.163442
56	2017	0.759178	-0.199275	0.2217	-0.0754165	0.419707	-0.125894
57	2017	0.882282	-0.493653	0.190996	0.0604116	0.511133	-0.15117
58	2017	0.756838	-0.148379	0.245496	-0.157206	0.415192	-0.111941
59	2017	0.786599	-0.15521	0.324543	-0.162057	0.31949	-0.113365
60	2017	0.836053	-0.17633	0.198452	-0.138818	0.393063	-0.112421
61	2017	0.614382	-0.0738231	0.258884	-0.0629027	0.380426	-0.116965
62	2017	0.836753	-0.491517	0.314157	-0.0281617	0.557903	-0.189135
63	2017	0.790322	-0.0949453	0.183239	-0.192816	0.442134	-0.127933
64	2017	0.653936	-0.0730068	0.374971	-0.136777	0.299578	-0.118701
65	2017	0.755294	-0.183962	0.288677	-0.0824311	0.340729	-0.118307
66	2017	0.690705	-0.0926274	0.303079	-0.0929661	0.293054	-0.101245
67	2017	0.867054	-0.378092	0.333988	-0.163656	0.529412	-0.188707
68	2017	0.75811	-0.339287	0.385279	-0.0737923	0.452223	-0.182533
69	2017	0.765494	-0.50638	0.249697	0.135222	0.494996	-0.139028
70	2017	0.796836	-0.225645	0.19872	-0.120322	0.503337	-0.152926
71	2017	0.787636	-0.260617	0.393344	-0.167424	0.414367	-0.167306
72	2017	102.555	-0.453492	0.133999	-0.114511	0.561011	-0.152553
73	2017	0.752296	-0.39719	0.505919	-0.0403948	0.383706	-0.204336
74	2017	0.941414	-0.404055	0.228205	-0.126403	0.494274	-0.133435
75	2017	0.730005	-0.381573	0.240479	0.0376652	0.522468	-0.149044
76	2017	0.813313	-0.174006	0.143165	-0.158754	0.476697	-0.100415
77	2017	0.811137	-0.281989	0.309074	-0.123711	0.431206	-0.145717

78	2017	0.912218	-0.351941	0.261656	-0.154247	0.503451	-0.171137
79	2017	0.749842	-0.195682	0.383892	-0.0876383	0.30821	-0.158623
80	2017	0.875896	-0.393904	0.321001	-0.130421	0.498251	-0.170823
81	2017	0.824723	-0.392721	0.458272	-0.0982567	0.389236	-0.181253
82	2017	0.78817	-0.175702	0.318286	-0.0963015	0.289553	-0.124006
83	2017	0.898574	-0.436439	0.295063	-0.048632	0.458829	-0.167394
84	2017	0.835905	-0.392408	0.327762	-0.0533824	0.44424	-0.162117
85	2017	0.732939	-0.179375	0.380759	-0.14393	0.373817	-0.164209
86	2017	0.874741	-0.356909	0.19215	-0.0708896	0.518249	-0.157341
87	2017	0.78208	-0.213296	0.340584	-0.150201	0.393257	-0.152423
88	2017	101.056	-0.455903	0.253874	-0.0747406	0.455618	-0.189411
89	2017	0.824034	-0.25156	0.243399	-0.0824177	0.386992	-0.120447
90	2017	0.975797	-0.347449	0.269766	-0.241739	0.536037	-0.192414
91	2017	0.838892	-0.333098	0.278499	-0.0883617	0.48047	-0.176401
92	2017	0.74051	-0.389182	0.37556	0.0140722	0.439797	-0.180757
93	2017	0.796236	-0.2973	0.348438	-0.110836	0.433924	-0.170461
94	2017	0.83884	-0.348463	0.201156	-0.0438356	0.51806	-0.165758
95	2017	0.772266	-0.26625	0.334928	-0.113465	0.439753	-0.167233
96	2017	0.616266	-0.519506	0.432385	0.206299	0.463618	-0.199061
97	2017	0.561635	-0.390821	0.355415	0.119075	0.4766	-0.121904
98	2017	0.819476	-0.312451	0.331665	-0.184056	0.529196	-0.183831
99	2017	0.876717	-0.390541	0.335587	-0.163289	0.522168	-0.180641
100	2017	0.976488	-0.391306	0.229504	-0.165065	0.529006	-0.178629
101	2017	0.577002	-0.461085	0.48937	0.170387	0.345466	-0.12114
102	2017	0.487744	-0.167828	0.447713	0.0218896	0.301863	-0.0913814
103	2017	0.721356	-0.502849	0.232679	0.080889	0.482438	-0.014513
104	2017	0.786711	-0.304601	0.25076	-0.0673245	0.470893	-0.136438
105	2017	0.634086	-0.204142	0.288867	0.00209878	0.371534	-0.0924432
106	2017	0.520138	-0.376818	0.284385	0.0773931	0.601972	-0.107069
107	2017	0.538514	-0.309471	0.454689	0.0304263	0.433388	-0.147546
108	2017	0.613458	-0.302867	0.562437	-0.00762864	0.319159	-0.184558
109	2017	0.679199	-0.401504	0.51101	0.00202089	0.36527	-0.155996
110	2017	0.658334	-0.287175	0.467195	-0.0233584	0.349548	-0.164544
111	2017	0.739108	-0.315326	0.257154	-0.0379446	0.474579	-0.117571
112	2017	0.668794	-0.326089	0.428227	0.0227088	0.345967	-0.139608
113	2018	0.725912	0.209151	0.464035	-0.12	0.46	-0.16
114	2018	0.760993	0.510027	0.526759	-0.18	0.53	-0.2
115	2018	0.794022	0.571803	0.492184	-0.19	0.49	-0.21
116	2018	0.561773	0.565772	0.486883	0.01	0.49	-0.19
117	2018	0.938621	0.615048	0.413961	-0.24	0.41	-0.24
118	2018	103.231	0.510659	0.50168	-0.24	0.5	-0.26
119	2018	0.640767	0.451832	0.5745	0	0.57	-0.18
120	2018	0.823563	0.466587	0.500438	-0.19	0.5	-0.19
121	2018	0.536936	0.29845	0.682002	0.18	0.68	-0.1
122	2018	0.532833	0.509727	0.545389	-0.15	0.55	-0.17
123	2018	0.855095	0.377855	0.558227	-0.18	0.56	-0.18
124	2018	0.866867	0.525508	0.491746	-0.17	0.49	-0.22
125	2018	0.339087	0.542978	0.57919	0.19	0.58	-0.16
126	2018	0.830003	0.616501	0.456869	-0.2	0.46	-0.23
127	2018	0.798336	0.55566	0.481897	-0.18	0.48	-0.24
128	2018	0.489882	0.416369	0.248033	0.09	0.25	-0.08
129	2018	0.651247	0.513242	0.191124	-0.06	0.19	-0.06
130	2018	0.437503	0.492544	0.194988	0.09	0.19	-0.08
131	2018	0.928625	0.462686	0.213802	-0.19	0.21	-0.16
132	2018	0.573459	0.358176	0.299119	0.11	0.3	-0.05
133	2018	0.512344	0.408698	0.222004	0.02	0.22	-0.07
134	2018	0.636508	0.535434	0.163046	0.04	0.16	-0.06
135	2018	0.732204	0.504899	0.1683	-0.18	0.17	-0.16
136	2018	0.905766	0.446902	0.200628	-0.19	0.2	-0.12
137	2018	0.445423	0.420504	0.275634	0.08	0.28	-0.07
138	2018	0.766042	0.511205	0.222272	0	0.22	-0.13
139	2018	0.346876	0.29763	0.427816	0.17	0.43	-0.04
140	2018	0.244534	0.426489	0.380404	0.09	0.38	-0.08
141	2018	0.705418	0.353996	0.339929	-0.17	0.34	-0.08
142	2018	0.583228	0.507557	0.270078	-0.22	0.27	-0.15
143	2018	0.702267	0.279455	0.367618	-0.18	0.37	-0.08
144	2018	0.629795	0.362602	0.330155	-0.12	0.33	-0.1
145	2018	0.668485	0.367153	0.299608	-0.15	0.3	-0.1
146	2018	0.566202	0.362414	0.325272	-0.12	0.33	-0.08
147	2018	0.691034	0.411456	0.258439	-0.2	0.26	-0.12
148	2018	0.752479	0.40154	0.204029	-0.24	0.2	-0.11

149	2018	0.568772	0.432034	0.268441	-0.13	0.27	-0.11
150	2018	0.806967	0.397036	0.413928	-0.18	0.41	-0.14
151	2018	0.821712	0.554066	0.275575	-0.25	0.28	-0.2
152	2018	0.759609	0.349814	0.441403	-0.13	0.44	-0.13
153	2018	0.533093	0.43994	0.372439	0.03	0.37	-0.09
154	2018	0.745521	0.420235	0.407859	-0.17	0.41	-0.14
155	2018	0.539856	0.297906	0.48512	0.16	0.49	-0.05
156	2018	0.475205	0.179327	0.620813	-0.06	0.62	-0.07
157	2018	0.876586	0.168138	0.977079	-0.43	0.98	-0.25
158	2018	0.642157	0.120611	0.991227	-0.09	0.99	-0.17
159	2018	0.935453	0.148692	0.909417	-0.26	0.91	-0.22
160	2018	0.603901	0.21609	0.939442	-0.05	0.94	-0.21
161	2018	0.632402	0.358838	0.85934	-0.27	0.86	-0.24
162	2018	0.768204	0.128847	100.431	-0.31	1	-0.18
163	2018	0.647732	0.200199	0.92999	-0.16	0.93	-0.19
164	2018	0.859547	0.232314	0.895058	-0.32	0.9	-0.24
165	2018	0.771726	0.248998	0.949435	-0.21	0.95	-0.18
166	2018	0.587688	0.171834	0.987674	-0.19	0.99	-0.19
167	2018	0.739339	0.194368	0.89925	-0.27	0.9	-0.2
168	2019	0.661322	0.268338	0.919001	-0.3	0.92	-0.23
169	2019	0.689303	0.33733	0.818163	-0.26	0.82	-0.27
170	2019	0.686478	0.164366	0.864608	-0.17	0.86	-0.19
171	2019	0.596168	0.266125	0.873778	-0.14	0.87	-0.17
172	2019	0.617639	0.425778	0.66929	-0.22	0.67	-0.23
173	2019	0.560057	0.277963	0.82741	-0.07	0.83	-0.18
174	2019	0.636056	0.357697	0.717512	-0.27	0.72	-0.17
175	2019	0.833667	0.240753	0.861269	-0.21	0.86	-0.24
176	2019	0.642067	0.315391	0.783582	-0.14	0.78	-0.2
177	2019	0.707312	0.245851	0.773579	-0.1	0.77	-0.18
178	2019	0.279361	0.535468	0.689136	-0.29	0.69	-0.23
179	2019	0.384032	0.386258	0.787814	-0.14	0.79	-0.18
180	2019	0.624676	0.0767368	116.667	-0.09	1.17	-0.19
181	2019	0.816545	0.228858	106.811	-0.23	1.07	-0.28
182	2019	0.76778	0.129558	116.832	-0.08	1.17	-0.21
183	2019	0.819565	-0.0331889	121.686	-0.18	1.22	-0.18
184	2019	0.629489	0.0219811	121.832	-0.05	1.22	-0.18
185	2019	0.752459	0.112262	115.992	-0.21	1.16	-0.24
186	2019	0.77852	0.044572	119.294	-0.19	1.19	-0.21
187	2019	102.944	0.0461857	114.622	-0.37	1.15	-0.27
188	2019	0.603683	0.284824	0.676922	-0.03	0.68	-0.13
189	2019	0.564445	0.168239	0.715954	-0.03	0.72	-0.09
190	2019	0.538046	0.19465	0.706579	-0.14	0.71	-0.09
191	2019	0.628151	0.232015	0.660223	-0.01	0.66	-0.11
192	2019	0.605443	0.203077	0.7174	-0.01	0.72	-0.11
193	2019	0.515826	-0.169725	0.202305	-0.143282	0.721455	-0.126579
194	2019	0.472121	-0.180338	0.154063	-0.0955101	0.760489	-0.110826
195	2019	0.472765	-0.224314	0.185329	-0.0715103	0.758836	-0.121106
196	2019	0.54212	-0.316289	0.0587262	-0.036268	0.840445	-0.0887348
197	2019	0.457724	-0.0666711	0.0440405	-0.165739	0.787526	-0.0568799
198	2019	0.933449	-0.292597	0.0759853	-0.28077	0.725809	-0.161876
199	2019	0.821894	-0.121057	0.25665	-0.378646	0.625826	-0.204667
200	2019	0.878658	-0.163737	0.082756	-0.341199	0.722505	-0.178983
201	2019	0.935812	-0.343122	-0.015968	-0.230802	0.769156	-0.115077
202	2019	0.801913	-0.256127	-0.0235311	-0.247441	0.844453	-0.119267
203	2019	0.724717	-0.168988	0.0131736	-0.281815	0.810283	-0.097371
204	2019	0.898975	-0.243531	-0.00950872	-0.337139	0.775618	-0.0844143
205	2019	0.81493	-0.208733	0.0554381	-0.261747	0.760887	-0.160775
206	2019	0.797576	-0.22599	0.0417209	-0.269306	0.753453	-0.0974547
207	2019	0.655524	-0.144416	0.142651	-0.265515	0.715393	-0.103636
208	2019	0.775845	-0.178388	0.108724	-0.303682	0.725897	-0.128396
209	2019	0.980014	-0.297283	0.00187774	-0.321721	0.807293	-0.170182
210	2019	0.623015	-0.156071	0.0679792	-0.188818	0.750727	-0.0968328
211	2019	0.785327	-0.395895	0.033545	-0.113752	0.788763	-0.0979892
212	2019	0.923546	-0.263664	0.0252846	-0.336014	0.794233	-0.143385
213	2019	0.838247	-0.24018	0.0222151	-0.285286	0.780058	-0.115054
214	2019	0.786476	-0.162492	0.0556604	-0.300297	0.76245	-0.141798
215	2019	0.824005	-0.29819	0.0826065	-0.273501	0.821716	-0.156637
216	2019	0.784186	-0.330204	0.0169595	-0.215504	0.851925	-0.107363
217	2019	0.546697	-0.382631	0.246383	0.0648841	0.655403	-0.130736
218	2019	0.646478	-0.262576	0.141858	-0.0781829	0.657706	-0.105284
219	2019	0.807011	-0.248177	0.118693	-0.218001	0.656864	-0.11639

220	2019	0.562516	-0.319059	0.0851278	0.0343655	0.760464	-0.123414
221	2019	0.825152	-0.396254	0.0604201	-0.111154	0.727535	-0.105698
222	2019	0.718971	-0.294899	0.0777658	-0.0844895	0.688026	-0.105375
223	2019	0.70241	-0.432339	0.162355	-0.05455	0.700691	-0.0785661
224	2019	0.684675	-0.426211	0.209789	-0.0335582	0.654229	-0.0889233
225	2019	0.607387	-0.431553	0.300694	0.0179548	0.619862	-0.114345
226	2019	0.764682	-0.471075	-0.0283118	-0.0532225	0.851097	-0.0631693
227	2019	0.74306	-0.484746	-0.192462	0.125976	0.860403	-0.0522313
228	2019	0.816012	-0.647478	-0.14059	0.0847995	0.951281	-0.0640241
229	2019	0.812609	-0.48465	-0.0466394	-0.147376	0.950742	-0.0846854
230	2019	0.904939	-0.468016	-0.140106	-0.135046	0.94331	-0.105081
231	2020	110.615	-0.576771	-0.0425454	-0.269391	0.908465	-0.125906
232	2020	103.571	-0.520044	-0.171524	-0.306215	105.376	-0.0916851
233	2020	102.014	-0.375581	0.033761	-0.360145	0.848369	-0.166543
234	2020	0.983355	-0.350307	-0.0830839	-0.293252	0.857887	-0.114599
235	2020	111.216	-0.481648	-0.102726	-0.271746	0.87986	-0.135902
236	2020	106.542	-0.536902	-0.0190517	-0.267418	0.908558	-0.150604
237	2020	105.648	-0.376143	-0.0267455	-0.303896	0.844019	-0.19371
238	2020	109.324	-0.40842	0.00640455	-0.307654	0.777995	-0.161564
239	2020	101.607	-0.356012	-0.0903048	-0.27343	0.840972	-0.137296
240	2020	0.956149	-0.358205	-0.0402027	-0.295462	0.868165	-0.130445
241	2020	0.947931	-0.374643	-0.0722732	-0.24959	0.880639	-0.132064

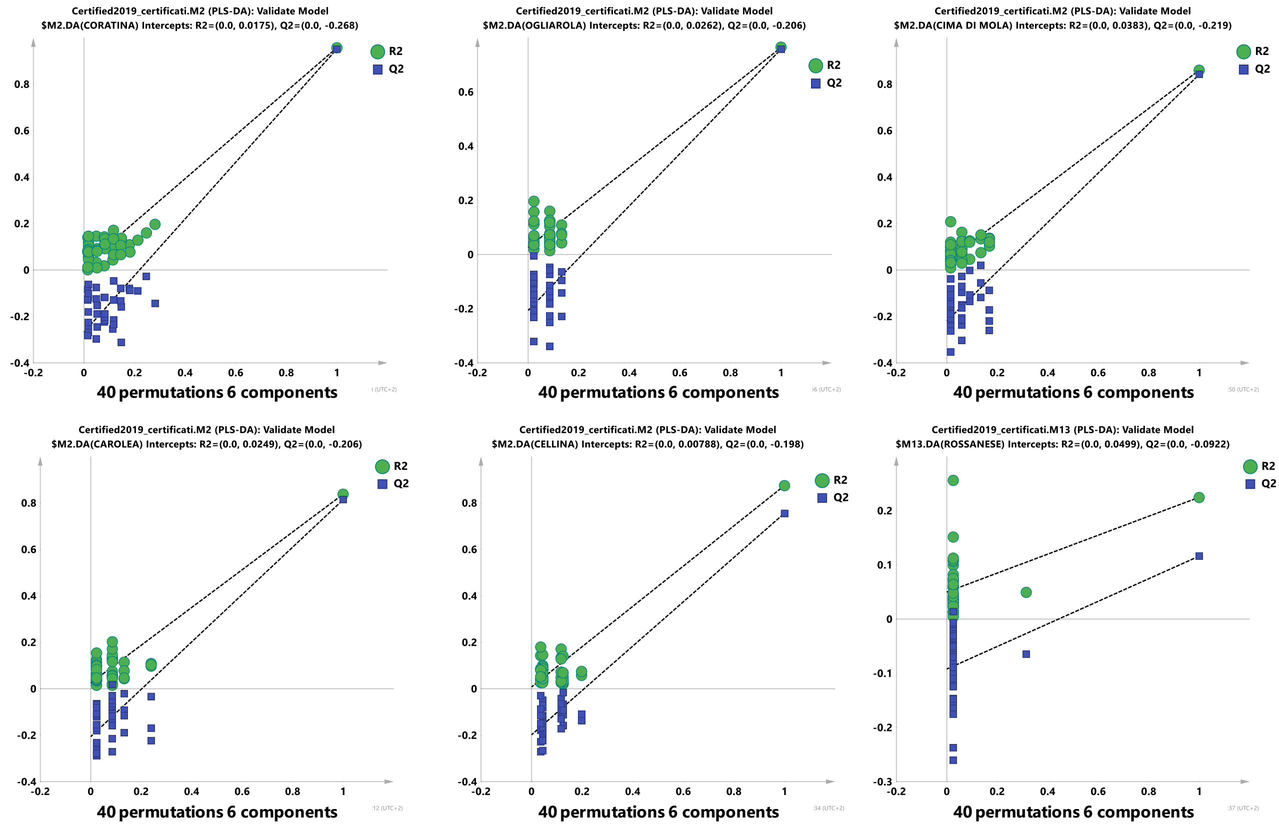

Figure S2. Permutation test performed with 40 cycles of random permutation of Y variables on PLS-DA models for Coratina, Ogliarola, Cima di Mola, Cellina, Carolea and Rossanese cultivars. The horizontal axis shows the correlation between the original and the permuted y. The vertical axis shows the values for R^2 (green line) and Q^2 (blue line).The intercept is a measure of the overfit.

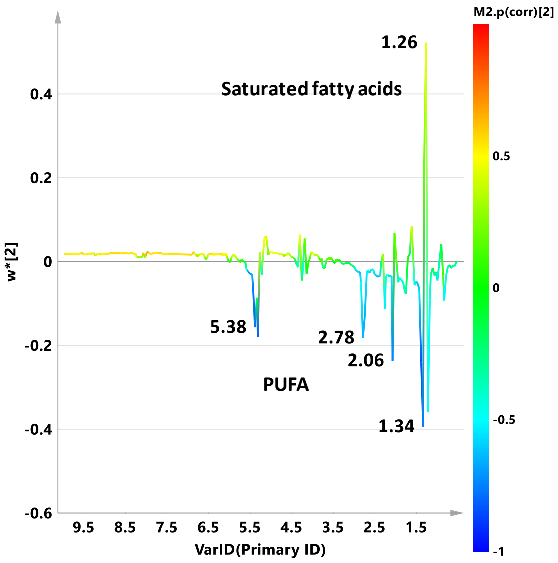

Figure S3. Line plot for the Figure 1a model, indicating the ^{1}H NMR chemical shifts of the signals, characteristic of specific metabolites, discriminating the classes along t[2] and coloured according to the correlation-scaled loading (*p(corr) ≥|0.5|). w*c[1] axis represented the weighted correlation vector

Table S2: Classification list for the observations (commercial 100% Italian EVOO blends from 2016/2017, 2017/2018, 2018/2019, 2019/2020 harvesting years) predicted on the OPLS-DA models built with zg and zg combined noesy bucket reduced spectra

**YPredPS[1](zg bucket).DA(CORATINA))**	**YPredPS[1](zg combined noesygpps bucket).DA(CORATINA)**	harvesting year
0.298997	0.328983	cert.orgins2017/18
0.413658	0.313404	cert.orgins2017/18
0.419764	0.333684	cert.orgins2017/18
0.441324	0.360899	cert.orgins2017/18
0.445345	0.43402	cert.orgins2017/18
0.45926	0.46398	cert.orgins2017/18
0.491423	0.415848	cert.orgins2017/18
0.491807	0.425292	cert.orgins2017/18
0.498116	0.412562	cert.orgins2017/18
0.507182	0.495448	cert.orgins2017/18
0.509911	0.412554	cert.orgins2017/18
0.512698	0.513004	cert.orgins2017/18
0.536798	0.431714	cert.orgins2017/18
0.576457	0.476194	cert.orgins2017/18
0.578853	0.451019	cert.orgins2017/18
0.590783	0.548933	cert.orgins2017/18
0.596339	0.368961	cert.orgins2017/18
0.598043	0.477588	cert.orgins2017/18
0.601232	0.481942	cert.orgins2017/18
0.612746	0.524825	cert.orgins2017/18
0.614196	0.456374	cert.orgins2017/18
0.617792	0.455499	cert.orgins2017/18
0.619712	0.534467	cert.orgins2017/18

0.619979	0.493615	cert.orgins2017/18
0.621872	0.479684	cert.orgins2017/18
0.623886	0.453689	cert.orgins2017/18
0.628022	0.47025	cert.orgins2017/18
0.640679	0.714725	cert.orgins2017/18
0.643154	0.593306	cert.orgins2017/18
0.645603	0.54311	cert.orgins2017/18
0.65208	0.541511	cert.orgins2017/18
0.658223	0.55372	cert.orgins2017/18
0.669575	0.601379	cert.orgins2017/18
0.671585	0.583824	cert.orgins2017/18
0.671998	0.448591	cert.orgins2017/18
0.672548	0.616476	cert.orgins2017/18
0.672548	0.616476	cert.orgins2017/18
0.678287	0.509127	cert.orgins2017/18
0.714229	0.76839	cert.orgins2017/18
0.714803	0.687486	cert.orgins2017/18
0.714897	0.520845	cert.orgins2017/18
0.72005	0.536201	cert.orgins2017/18
0.731937	0.479492	cert.orgins2017/18
0.733969	0.579547	cert.orgins2017/18
0.735332	0.590909	cert.orgins2017/18
0.751356	0.689421	cert.orgins2017/18
0.751356	0.689421	cert.orgins2017/18
0.761116	0.631876	cert.orgins2017/18
0.766418	0.586888	cert.orgins2017/18
0.767081	0.658426	cert.orgins2017/18
0.778099	0.578897	cert.orgins2017/18
0.790901	0.623797	cert.orgins2017/18
0.812449	0.608719	cert.orgins2017/18
0.817767	0.763242	cert.orgins2017/18
0.825698	0.590886	cert.orgins2017/18
0.828944	0.665451	cert.orgins2017/18
0.834179	0.692376	cert.orgins2017/18
0.834907	0.723848	cert.orgins2017/18
0.835461	0.693492	cert.orgins2017/18
0.836663	0.808705	cert.orgins2017/18
0.836663	0.808705	cert.orgins2017/18
0.846787	0.812119	cert.orgins2017/18
0.848297	0.697121	cert.orgins2017/18
0.874552	0.877178	cert.orgins2017/18
0.897794	0.61626	cert.orgins2017/18
0.904033	0.732482	cert.orgins2017/18
0.91955	0.806003	cert.orgins2017/18
0.91955	0.806003	cert.orgins2017/18
0.502988	0.452598	cert.origins2016
0.524539	0.384866	cert.origins2016

0.580614	0.409615	cert.origins2016
0.604002	0.400294	cert.origins2016
0.615709	0.646373	cert.origins2016
0.617056	0.598459	cert.origins2016
0.621424	0.648763	cert.origins2016
0.621872	0.479684	cert.origins2016
0.645484	0.535867	cert.origins2016
0.658501	0.696876	cert.origins2016
0.658501	0.696876	cert.origins2016
0.677633	0.64702	cert.origins2016
0.691078	0.595241	cert.origins2016
0.714803	0.687486	cert.origins2016
0.725602	0.758404	cert.origins2016
0.73645	0.56827	cert.origins2016
0.742483	0.667432	cert.origins2016
0.745871	0.71341	cert.origins2016
0.745871	0.71341	cert.origins2016
0.788774	0.733119	cert.origins2016
0.792187	0.683219	cert.origins2016
0.80486	0.735365	cert.origins2016
0.814978	0.822183	cert.origins2016
0.8195	0.55544	cert.origins2016
0.829538	0.689362	cert.origins2016
0.841079	0.887113	cert.origins2016
0.851062	0.822908	cert.origins2016
0.851062	0.822908	cert.origins2016
0.87136	0.720026	cert.origins2016
0.88944	0.887969	cert.origins2016
0.905418	0.754382	cert.origins2016
0.915586	0.657146	cert.origins2016
0.918053	0.689802	cert.origins2016
0.923267	0.731913	cert.origins2016
0.933204	0.911275	cert.origins2016
0.951089	0.769365	cert.origins2016
0.982439	0.888026	cert.origins2016
0.999752	0.817584	cert.origins2016
0.252818	0.262401	cert.origins2018/19
0.279503	0.255105	cert.origins2018/19
0.433332	0.434421	cert.origins2018/19
0.438552	0.357999	cert.origins2018/19
0.446777	0.498299	cert.origins2018/19
0.453654	0.244849	cert.origins2018/19
0.456478	0.330113	cert.origins2018/19
0.45926	0.46398	cert.origins2018/19
0.46414	0.418447	cert.origins2018/19
0.465578	0.482788	cert.origins2018/19
0.468566	0.42195	cert.origins2018/19

0.46971	0.361855	cert.origins2018/19
0.472594	0.356753	cert.origins2018/19
0.476187	0.462768	cert.origins2018/19
0.479027	0.477862	cert.origins2018/19
0.486067	0.431685	cert.origins2018/19
0.486684	0.384856	cert.origins2018/19
0.507076	0.521143	cert.origins2018/19
0.514607	0.460232	cert.origins2018/19
0.516704	0.412405	cert.origins2018/19
0.517985	0.540923	cert.origins2018/19
0.527549	0.418211	cert.origins2018/19
0.531773	0.411494	cert.origins2018/19
0.537397	0.260424	cert.origins2018/19
0.538425	0.380488	cert.origins2018/19
0.545302	0.416382	cert.origins2018/19
0.555603	0.515145	cert.origins2018/19
0.558041	0.307786	cert.origins2018/19
0.562879	0.447441	cert.origins2018/19
0.574428	0.446421	cert.origins2018/19
0.577105	0.561565	cert.origins2018/19
0.584011	0.425138	cert.origins2018/19
0.590552	0.509733	cert.origins2018/19
0.61166	0.467133	cert.origins2018/19
0.612746	0.524825	cert.origins2018/19
0.617588	0.421254	cert.origins2018/19
0.635505	0.611185	cert.origins2018/19
0.637974	0.624441	cert.origins2018/19
0.640038	0.657077	cert.origins2018/19
0.64052	0.436042	cert.origins2018/19
0.64253	0.393051	cert.origins2018/19
0.645426	0.588903	cert.origins2018/19
0.647242	0.528771	cert.origins2018/19
0.665265	0.520137	cert.origins2018/19
0.675731	0.497916	cert.origins2018/19
0.681529	0.61004	cert.origins2018/19
0.681529	0.61004	cert.origins2018/19
0.691933	0.653249	cert.origins2018/19
0.700559	0.478788	cert.origins2018/19
0.703844	0.67296	cert.origins2018/19
0.719112	0.446476	cert.origins2018/19
0.727241	0.537456	cert.origins2018/19
0.727681	0.674858	cert.origins2018/19
0.757595	0.623017	cert.origins2018/19
0.765825	0.526008	cert.origins2018/19
0.780398	0.566236	cert.origins2018/19
0.783339	0.473351	cert.origins2018/19
0.784436	0.594039	cert.origins2018/19

0.792582	0.644122	cert.origins2018/19
0.799357	0.682514	cert.origins2018/19
0.800729	0.710304	cert.origins2018/19
0.812731	0.56294	cert.origins2018/19
0.819057	0.578904	cert.origins2018/19
0.822246	0.531525	cert.origins2018/19
0.831142	0.705205	cert.origins2018/19
0.843241	0.685568	cert.origins2018/19
0.846264	0.700138	cert.origins2018/19
0.861814	0.996858	cert.origins2018/19
0.863911	0.851431	cert.origins2018/19
0.874552	0.877178	cert.origins2018/19
0.877105	0.592417	cert.origins2018/19
0.880087	0.598749	cert.origins2018/19
0.891012	0.681256	cert.origins2018/19
0.902271	0.681936	cert.origins2018/19
0.90391	0.756845	cert.origins2018/19
0.926851	0.684514	cert.origins2018/19
0.933204	0.911275	cert.origins2018/19
0.962718	0.922371	cert.origins2018/19
0.353531	0.363417	cert.origins2019/20
0.478639	0.527252	cert.origins2019/20
0.478639	0.527252	cert.origins2019/20
0.489542	0.405318	cert.origins2019/20
0.495069	0.468557	cert.origins2019/20
0.49893	0.427053	cert.origins2019/20
0.512698	0.513004	cert.origins2019/20
0.518487	0.430809	cert.origins2019/20
0.531842	0.506168	cert.origins2019/20
0.573088	0.560339	cert.origins2019/20
0.579588	0.581319	cert.origins2019/20
0.579588	0.581319	cert.origins2019/20
0.581809	0.522082	cert.origins2019/20
0.593029	0.595713	cert.origins2019/20
0.596343	0.630403	cert.origins2019/20
0.596456	0.51068	cert.origins2019/20
0.597879	0.468026	cert.origins2019/20
0.605876	0.469795	cert.origins2019/20
0.617056	0.598459	cert.origins2019/20
0.637974	0.624441	cert.origins2019/20
0.638925	0.508691	cert.origins2019/20
0.683901	0.407443	cert.origins2019/20
0.692225	0.366993	cert.origins2019/20
0.697258	0.557383	cert.origins2019/20
0.697636	0.636846	cert.origins2019/20
0.718317	0.633392	cert.origins2019/20
0.728872	0.760422	cert.origins2019/20

0.733715	0.523434	cert.origins2019/20
0.747966	0.578833	cert.origins2019/20
0.765447	0.573604	cert.origins2019/20
0.770357	0.723151	cert.origins2019/20
0.779704	0.628886	cert.origins2019/20
0.805253	0.763011	cert.origins2019/20
0.809457	0.896191	cert.origins2019/20
0.823395	0.641154	cert.origins2019/20
0.830881	0.721247	cert.origins2019/20
0.847104	0.784142	cert.origins2019/20
0.848727	0.796204	cert.origins2019/20
0.850474	0.804038	cert.origins2019/20
0.860611	0.761084	cert.origins2019/20
0.860611	0.761084	cert.origins2019/20
0.873602	0.918588	cert.origins2019/20
0.873602	0.918588	cert.origins2019/20
0.87879	0.974634	cert.origins2019/20
0.911853	0.92538	cert.origins2019/20
0.912298	0.941264	cert.origins2019/20
0.914461	0.784735	cert.origins2019/20
0.962718	0.922371	cert.origins2019/20
0.982439	0.888026	cert.origins2019/20

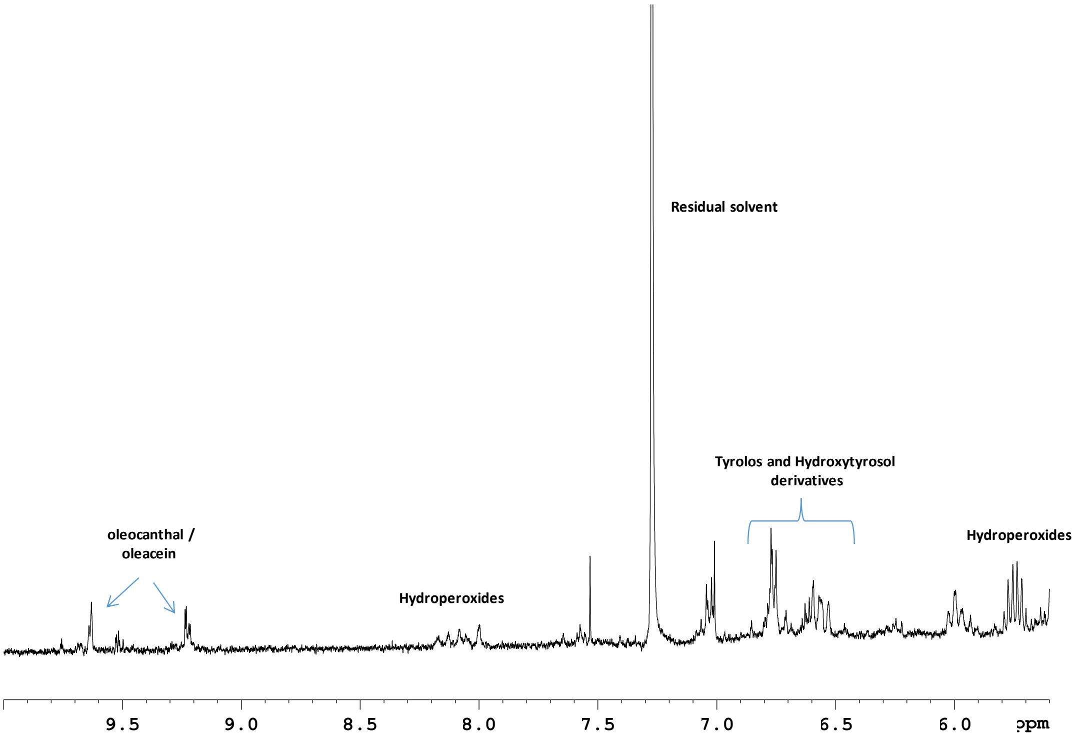

Figure S4 Representative noesygpps ^{1}H NMR spectra of EVOO sample. Main metabolites are indicated

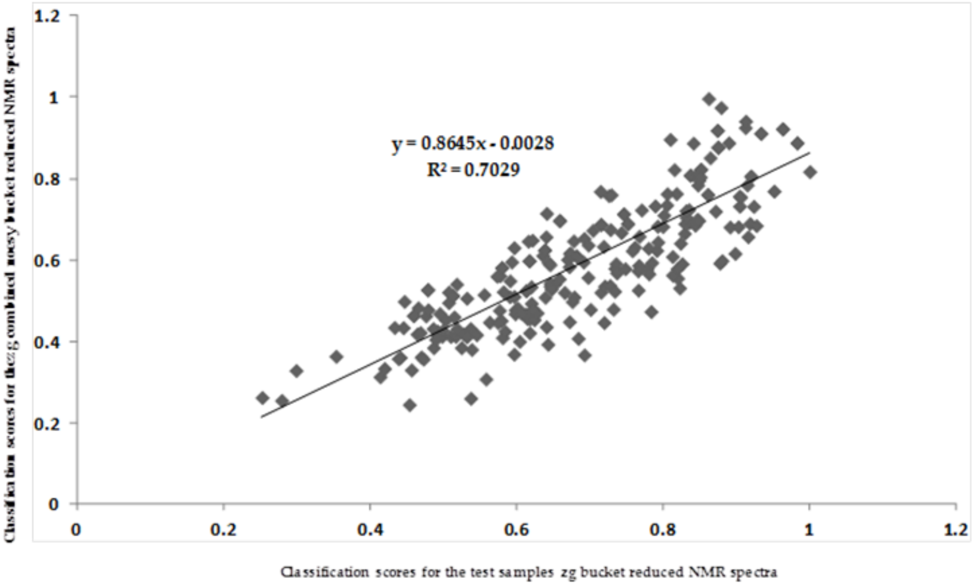

Figure S5. Relationship between classification scores of the commercial blend samples predicted on the bucket reduced combined zg - noesy NMR spectra.
